# Exploring BSEP inhibition-mediated toxicity with a mechanistic model of drug-induced liver injury

**DOI:** 10.3389/fphar.2014.00240

**Published:** 2014-11-07

**Authors:** Jeffrey L. Woodhead, Kyunghee Yang, Scott Q. Siler, Paul B. Watkins, Kim L. R. Brouwer, Hugh A. Barton, Brett A. Howell

**Affiliations:** ^1^The Hamner-UNC Institute for Drug Safety Sciences, The Hamner Institutes for Health SciencesResearch Triangle Park, NC, USA; ^2^Division of Pharmacotherapy and Experimental Therapeutics, UNC-Eshelman School of Pharmacy, University of North Carolina at Chapel HillChapel Hill, NC, USA; ^3^Pharmacokinetics, Dynamics, and Metabolism, Worldwide Research and Development, Pfizer, Inc. GrotonCT, USA

**Keywords:** BSEP inhibition, bile acids and salts, drug-induced liver injury, mechanistic modeling, bosentan, CP-724,714, population variability

## Abstract

Inhibition of the bile salt export pump (BSEP) has been linked to incidence of drug-induced liver injury (DILI), presumably by the accumulation of toxic bile acids in the liver. We have previously constructed and validated a model of bile acid disposition within DILIsym®, a mechanistic model of DILI. In this paper, we use DILIsym® to simulate the DILI response of the hepatotoxic BSEP inhibitors bosentan and CP-724,714 and the non-hepatotoxic BSEP inhibitor telmisartan in humans in order to explore whether we can predict that hepatotoxic BSEP inhibitors can cause bile acid accumulation to reach toxic levels. We also simulate bosentan in rats in order to illuminate potential reasons behind the lack of toxicity in rats compared to the toxicity observed in humans. DILIsym® predicts that bosentan, but not telmisartan, will cause mild hepatocellular ATP decline and serum ALT elevation in a simulated population of humans. The difference in hepatotoxic potential between bosentan and telmisartan is consistent with clinical observations. However, DILIsym® underpredicts the incidence of bosentan toxicity. DILIsym® also predicts that bosentan will not cause toxicity in a simulated population of rats, and that the difference between the response to bosentan in rats and in humans is primarily due to the less toxic bile acid pool in rats. Our simulations also suggest a potential synergistic role for bile acid accumulation and mitochondrial electron transport chain (ETC) inhibition in producing the observed toxicity in CP-724,714, and suggest that CP-724,714 metabolites may also play a role in the observed toxicity. Our work also compares the impact of competitive and noncompetitive BSEP inhibition for CP-724,714 and demonstrates that noncompetitive inhibition leads to much greater bile acid accumulation and potential toxicity. Our research demonstrates the potential for mechanistic modeling to contribute to the understanding of how bile acid transport inhibitors cause DILI.

## Introduction

Inhibition of the bile salt export pump (BSEP) by a drug has been implicated as a risk factor for the drug's potential to cause drug-induced liver injury (DILI) (Dawson et al., 2011; Morgan et al., 2013). Several high-profile DILI-causing drugs, for example troglitazone (Funk et al., 2001; Smith, 2003), bosentan (Fattinger et al., 2001; Eriksson et al., 2011), and nefazodone (Kostrubsky et al., 2006), have been shown to be BSEP inhibitors. Furthermore, prediction of toxicity by these molecules has been uneven; for example, neither bosentan nor troglitazone displayed toxicity in animal models (Leslie et al., 2007; Lauer et al., 2009). Predictions involving hepatobiliary transporter IC_50_ values in *in vitro* assays have shown better predictive ability (Dawson et al., 2011; Morgan et al., 2013). Improving the ability to predict the frequency and severity of DILI with BSEP inhibitors will allow those involved in drug development to better judge the risk involved with moving a drug in development into the clinic or beyond early-stage clinical trials.

DILIsym® is a multi-scale mechanistic model incorporating numerous functions of the liver and disruptions of the function with the goal of predicting the DILI potential of drugs at various stages in the development process (Howell et al., 2012, 2014; Woodhead et al., 2012; Shoda et al., 2014). Previously, we have constructed and validated a model of bile acid homeostasis and transporter inhibition within DILIsym® (Woodhead et al., 2014). We found that inhibiting BSEP could lead to significant increases in bile acid concentrations in the liver, and that the effects of bile acid transporter inhibitors should be considered on a simulated population as well as on a single baseline individual. We have expanded that model to include a representation of bile acid-mediated toxicity based on experiments performed by Yang et al. (2013). In that work, we constructed a relationship between intracellular bile acid concentration and cellular ATP. We used this relationship to predict cellular necrosis using the existing relationship between ATP and cell death in DILIsym®. This model has been used previously to effectively predict the frequency and timing of ALT elevations observed with troglitazone in clinical trials, and has predicted the difference between troglitazone and the similar non-toxic drug pioglitazone (Yang et al., 2014).

In the present work, we use this model of bile acid-mediated cytotoxicity to model the observed hepatotoxicity of bosentan, telmisartan, and CP-724,714. Bosentan is a currently marketed medication for pulmonary arterial hypertension that carries a black-box warning for hepatotoxicity. In clinical trials a dose of 1000 mg/day of bosentan caused between 8 and 18% of individuals to experience increases in serum ALT greater than 3-fold (Fattinger et al., 2001). Bosentan has also been shown to be a relatively potent BSEP inhibitor. Telmisartan, an angiotensin II receptor agonist used in hypertension treatment, is also a relatively potent BSEP inhibitor, but has not been reported to cause hepatotoxicity in humans (Morgan et al., 2013). CP-724,714 is an anti-cancer drug that was terminated from development after Phase II clinical trials revealed liver signals (Guo et al., 2008). CP-724,714 has been shown to inhibit multiple transporters, including BSEP, in addition to being a mitochondrial toxin (Feng et al., 2009). In this report we explore the DILIsym® software's (version 2C) predictions for the toxicity of bosentan in humans, and the lack of toxicity of bosentan in rats and telmisartan in humans. We will also test several theories about the toxicity of CP-724,714 in order to suggest potential viable explanations for the observed toxicity in early clinical trials.

## Methods

**Figure 1:**
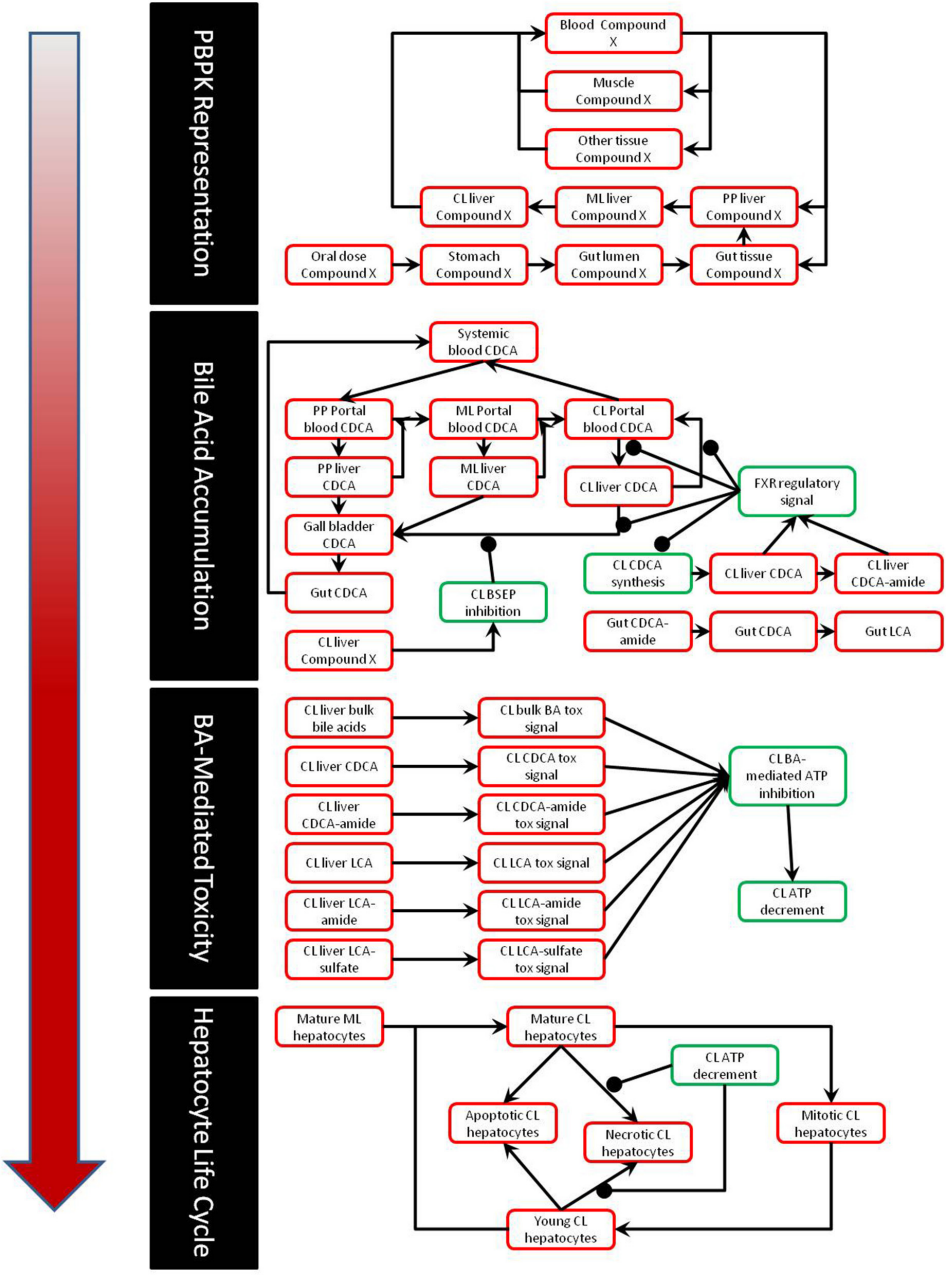
**Partial diagram of the portions of DILIsym® used for this paper, including the PBPK representation, representation of bile acid homeostasis and accumulation, link between bile acid concentrations and toxicity, and the hepatocyte life cycle**.

The construction and validation of the bile acid homeostasis model in DILIsym® is described in a previous paper (Woodhead et al., 2014). DILIsym® models the synthesis and enterohepatic recirculation of two main potentially toxic bile acids, chenodeoxycholic acid (CDCA) and lithocholic acid (LCA), and their amide (and sulfate in the case of LCA) conjugates. DILIsym® describes the intrahepatic accumulation of these toxic bile acids as well as the concentrations in the gallbladder, portal blood, gut lumen, and systemic blood. Concentrations of bile acid transporter inhibitors are modeled using a physiologically-based pharmacokinetic model (PBPK) described in depth in previous papers (Howell et al., 2012; Woodhead et al., 2012). The bile acid concentrations are linked to ATP decline as described below; the effect of ATP decline on eventual hepatocyte necrosis is described by a model of the hepatocyte life cycle also described in previous papers (Howell et al., 2012; Woodhead et al., 2012; Shoda et al., 2014). A diagram of the interaction between the various submodels of DILIsym® employed in this paper can be seen in Figure 1.

**Figure 2:**
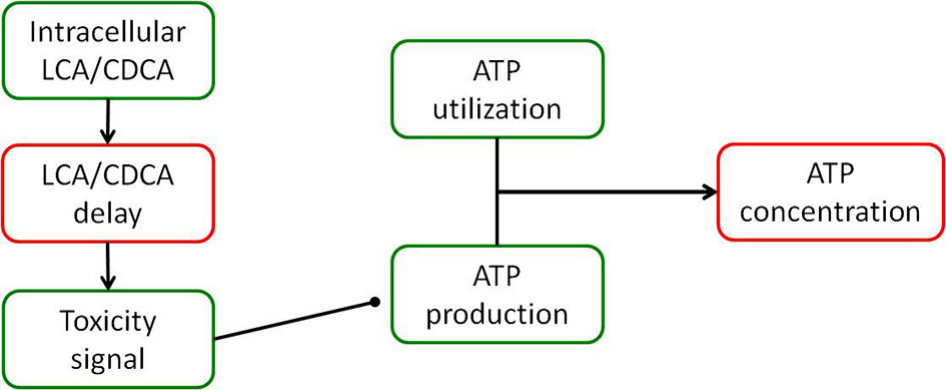
**Diagram of the model of ATP turnover used to parameterize the hepatocellular toxic response to accumulation of bile acids**. The model was set up to accept the experimentally measured intracellular concentration of each bile acid, and parameters were fit to the model in order to fit the experimentally observed intracellular ATP levels for each bile acid exposure.

The DILIsym® model of bile acid toxicity is based on *in vitro* experiments comparing the intracellular level of LCA and CDCA to cellular ATP levels (Yang et al., 2013). In order to construct the connection between bile acid accumulation and toxicity, a small model of the DILIsym® ATP turnover model was constructed (Yang et al., 2013); a diagram of this model is shown in Figure 2. The relationship between intracellular ATP and intracellular bile acids was modeled by the following equation:

$$\frac{d[ATP]}{dt}=k_{usage}-k_{synth}S$$

where *k*_*usage*_ is the rate of *ATP* usage in the cell, k_*synth*_ is the rate of ATP synthesis in the cell, and *S* is the signal for ATP synthesis inhibition by bile acids. *S* has Hill type behavior and is given by the following equation:

$$S=\frac{1}{1+\frac{V_{\max,S}{\left[BA\right]}_{delay}^{H}}{K_{m,S}^{H}+{\left[BA\right]}_{delay}^{H}}}$$

where *V*_max,*S*_ is the maximum denominator for the signal, *K*_*m,S*_ is the Michaelis constant for the signal, and *H* is the Hill coefficient for the signal. [*BA*]_*delay*_ is the delayed intracellular bile acid concentration, given by the standard delay equation:

$$\frac{d{\left[BA\right]}_{delay}}{dt}=\tau \left([BA]-{\left[BA\right]}_{delay}\right)$$

The delay constant τ, *V*_max,*S*_, *K*_*m,S*_, and *H* were the parameters that were fitted to the ATP time course from the experiment. These parameters were then applied to the intracellular bile acid concentrations modeled in DILIsym®. We applied the parameters for the unconjugated bile acids measured in the experiments to the conjugated bile acids in DILIsym®; for example, we used the unconjugated LCA parameters to describe the toxicity of LCA amide and sulfate conjugates. While some data exist describing the relationship between intracellular amide- and sulfate-conjugated bile acids and toxicity (Chatterjee et al., 2013), these are not enough to justify using different toxicity parameters for conjugated and unconjugated bile acids; this is an area of potential refinement for DILIsym®.

**Figure 3:**
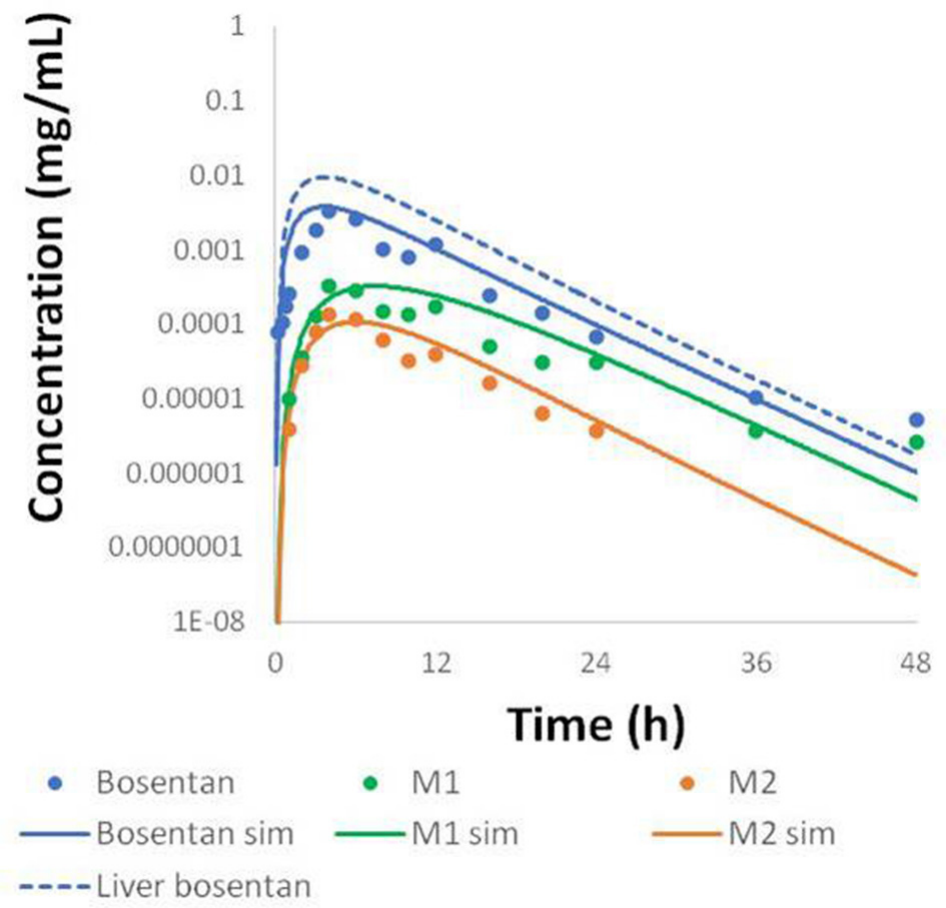
**Simulated plasma concentration of bosentan in humans and its two metabolites after a 500 mg oral dose compared to data from Weber et al. (1996)**.

**Figure 4:**
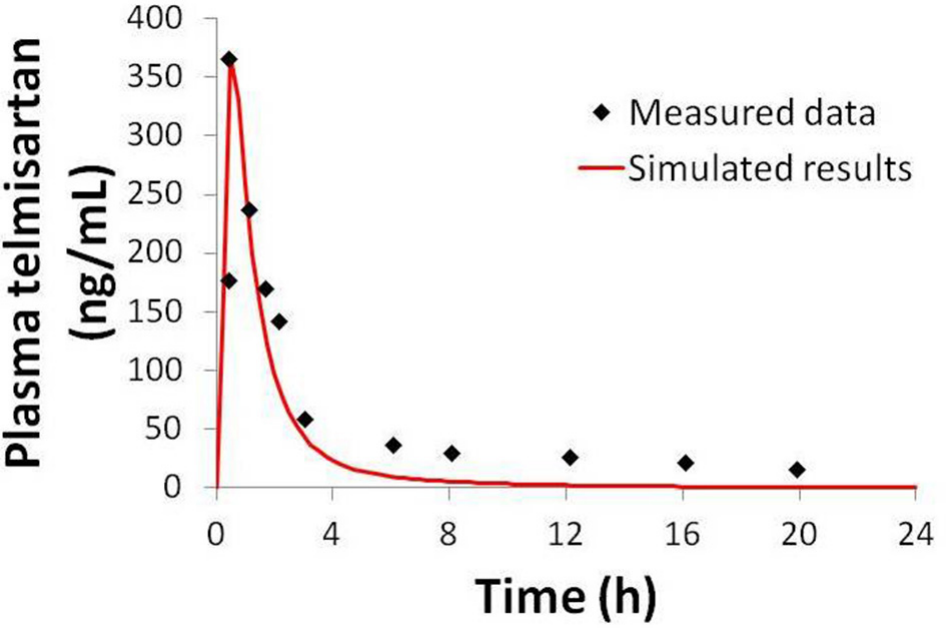
**Simulated plasma telmisartan concentration in humans after a single 80 mg oral dose compared to data from Stangier et al. (2000)**.

**Figure 5:**
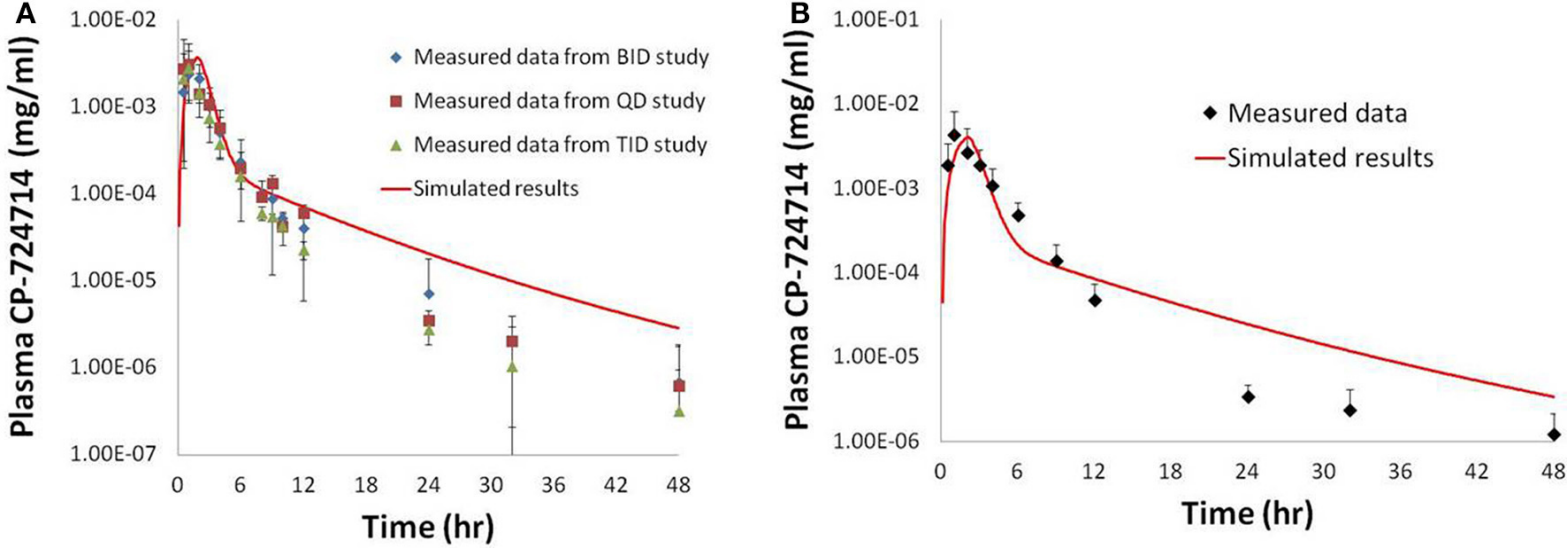
**Simulated plasma human concentrations of CP-724,714 after an oral dose of 250 mg (A) and 400 mg (B) compared to data from Munster et al. (2007)**. The 250 mg dose data represented in this figure are a compilation of the results from the first dose of several different dosing protocols.

**Figure 6:**
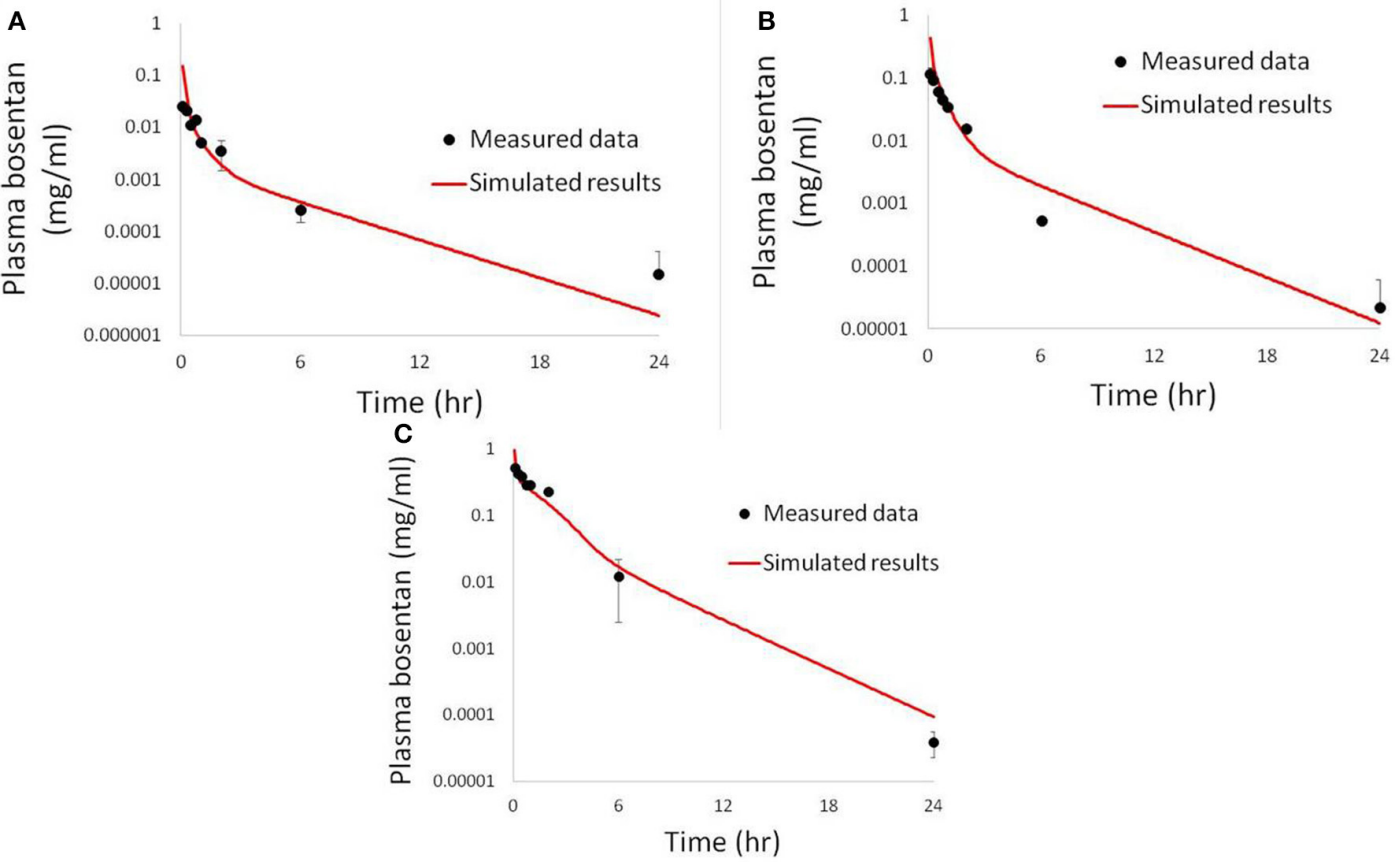
**Simulated plasma bosentan concentrations in rats after an intravenous oral dose of 10 mg (A), 30 mg (B), and 100 mg (C) compared to data provided by Amgen, Inc. (Thousand Oaks, CA)**.

Physiologically-based pharmacokinetic (PBPK) models were constructed for bosentan, telmisartan, and CP-724,714 in humans using available *in vivo* time course data (Weber et al., 1996; Stangier et al., 2000; Munster et al., 2007). For simulations where bosentan metabolism was impaired, the *V*_max_ for the metabolism of both metabolites was represented as 10% of their baseline value. Both bosentan and telmisartan are active transport substrates; the active transport parameters for both were adapted for use in DILIsym® from Ménochet et al. (2012). The bosentan PBPK model includes the major and minor metabolite, with metabolism parameters based on published *in vitro* metabolism data (Ubeaud et al., 1995); these were included because the minor metabolite of bosentan also inhibits BSEP (Fattinger et al., 2001). The major metabolite of telmisartan, a glucuronide, has not been shown to inhibit BSEP and so was not included in the PBPK model. CP-724,714's complex metabolism was not modeled explicitly; however, a single metabolite compartment in the liver was included in order to test the hypothesis of whether metabolite accumulation could explain CP-724,714 toxicity. PBPK model results for human bosentan are shown in Figure 3; those for human telmisartan are shown in Figure 4; and those for human CP-724,714 in Figure 5. For bosentan in rats, data did not exist in the literature. Data provided to us from Amgen, Inc. (Thousand Oaks, CA) were used to parameterize the bosentan rat PBPK model shown in Figure 6.

Inhibition constants for bosentan were taken from published research that found *K*_*i*_ values for both bosentan (12 μM) and the minor metabolite of bosentan (8.5 μM) (Fattinger et al., 2001). The inhibition of BSEP by bosentan and its metabolite were modeled as noncompetitive in agreement with the data. These data are from rat Bsep vesicles; we assigned the same *K*_*i*_ value to humans, as there is evidence suggesting that rat Bsep and human BSEP are inhibited with similar potency by bosentan (Mano et al., 2007). Bile acid uptake inhibition constants for bosentan were taken from work done by Leslie et al. (2007). The telmisartan *K*_*i*_ was approximated using the IC_50_ value reported in the literature (Morgan et al., 2013). A list of *K*_*i*_ values used in our simulations is included in Table 1. In addition, bosentan is a potent inducer of its own uptake into the liver and metabolism (Dingemanse and van Giersbergen, 2004); these effects are included in our PBPK model. It is important to note that for each drug, only the parameters related to drug pharmacokinetics and transporter inhibition were changed; all other remaining parameters were not changed from drug to drug.

**Table 1:** **Inhibition constants used for the compounds in the simulations conducted for this paper**.

**Compound**	**Species**	**Transporter**	***K*_*i*_**	**Mode**	**References**
Bosentan	Rat	BSEP	12 μM	Noncompetitive	Fattinger et al., 2001
Bosentan minor metabolite	Rat	BSEP	8.5 μM	Noncompetitive	Fattinger et al., 2001
Bosentan	Rat	NTCP	0.23 μM	Noncompetitive	Leslie et al., 2007
Bosentan	Human	NTCP	18 μM	Competitive	Leslie et al., 2007
Telmisartan	Human	BSEP	16 μM^*^	N/A	Morgan et al., 2013
CP-724,714	Human	BSEP	7.45 μM^*^	N/A	Novel data

* Denotes that the K_i_ was approximated using the listed IC_50_ value for which mode of inhibition was not determined.

For CP-724,714 IC_50_ values, experiments were run in rat Bsep, dog Bsep, and human BSEP-expressing vesicles. CP-724,714 was synthesized by Pfizer, Inc. (Groton, CT), while radiochemicals were purchased from Perkin Elmer (Perkin Elmer, Waltham MA). Other chemicals were purchased from Sigma-Aldrich (Sigma-Aldrich, St Louis, MO) or were of analytical grade. Membrane vesicles were obtained from Sf9 cells not expressing and expressing human BSEP (Solvo Biotechnology, Szeged, Hungary), rat Bsep (AB Life Technologies, Waltham, MA), or dog Bsep (AB Life Technologies).

CP-724,714 was incubated (2 or 5 min) with membrane vesicle preparations (total protein: 50 μg/well) and the probe substrate, taurocholate (2 μM). Serial dilutions of CP-724,714 (30 mM stock, 10 mM, 3.16 mM, 1 mM, 316 μM, 100 μM, 31.6 μM, 10 μM) were prepared in DMSO. Incubations in duplicate were carried out in the absence or presence of 4 mM ATP to distinguish between transporter mediated uptake and passive diffusion into the vesicles. CP-724,714 was added to the reaction mixture in 0.75 μl of solvent (1% of the final incubation volume). Glyburide (100 – 0.1 μM) was used as the positive control inhibitor. Reaction mixtures were pre-incubated for 10 min at 37°C. Reactions were started by the addition of 25 μl of 12 mM MgATP (or AMP, as disodium salt, for background controls), pre-incubated separately. Reactions were stopped by the addition of 200 μl of ice-cold washing buffer and immediate filtration via glass fiber filters mounted to a 96-well plate (filter plate). The filters were washed, dried and the amount of substrate inside the filtered vesicles determined by liquid scintillation. Maximal observed inhibition ranged between 94.8 and 99.8% for CP-724,714. Further information on this method can be found in the literature (Kis et al., 2009).

Bosentan dosing in humans was simulated as a 500-mg twice-daily dose for 30 days. The doses were given once every 12 h; this is one of the dosing regimens in the clinical trials that demonstrated toxicity as reported by Fattinger et al. (2001). Telmisartan dosing was simulated as a 50, 3000, or 12,000-mg dose once daily for 30 days; common clinical dosing regimens range from 40 to 80 mg per day (Meredith, 1999). CP-724,714 was dosed three times daily, or once every 8 h. A range of CP-724,714 doses were simulated in order to cover the range of exposure levels reported by Guo et al. (2008). Three simulated meals per day were included in all human simulations, and simulated to occur concurrent with drug dosing. Bosentan dosing in rats was simulated as a 50 mg/kg dose once daily.

DILIsym® contains simulated populations, or SimPops™, that represent a plausible range of variability in key model parameters. The human SimPops™ and rat SimPops™ used in the course of these simulations contain variability in several bile acid transport parameters and are described in our previous work (Woodhead et al., 2014; Yang et al., 2014). There are 331 individuals in the human SimPops™ and 191 individuals in the rat SimPops™; although the numbers of individuals are different, both the human and the rat SimPops™ are designed to account for the entire plausible range in variability in bile acid transport parameters and bile acid profiles observed in a sample population (Woodhead et al., 2014). Pharmacokinetic variability in bosentan disposition was also included in one SimPops™ simulation; for this population, a normal distribution around four variables (oral absorption coefficient, liver uptake *V*_max_, and major and minor metabolite formation *V*_max_) was superimposed upon the existing small human SimPops™. The range of these parameter values was based on a known range of variability for hepatobiliary transporters (Meier et al., 2006) and upon previous simulations where pharmacokinetic variability was considered (Woodhead et al., 2012).

## Results

Results from the SimPops™ analysis with bosentan are presented in Table 2. When we simulated bosentan in the human SimPops™ using the baseline assumptions (noncompetitive inhibition, no basolateral inhibition, normal metabolism), 1 individual out of 331 developed ALT elevations greater than 3-fold above the baseline value. When potential variability in pharmacokinetics was incorporated into the SimPops™, the predicted incidence rate rose to 3 out of 331. While this successfully predicts the potential toxicity of bosentan, the incidence rate is well below the incidence rate of 8–18% observed during the clinical trials (Fattinger et al., 2001).

**Table 2:** **SimPops™ simulation results for simulated ALT elevations caused by bosentan and telmisartan**.

**SimPops simulation**	**Number of ALT > 3 ×**	**Percentage**
Bosentan – baseline	1	0.302%
Bosentan – PK/toxicity variability	3	0.906%
Bosentan – 10× lower metabolism	8	2.42%
Bosentan – basolateral transporter inhibition	4	1.21%
Bosentan – rat	0	0
Telmisartan – competitive	0	0
Telmisartan – noncompetitive, 50 mg/day	0	0
Telmisartan – noncompetitive, 3000 mg/day	0	0
Telmisartan – noncompetitive, 12,000 mg/day	1	0.302%

A genetic polymorphism that limits CYP metabolism has been shown to correlate with an increased rate of toxicity from bosentan exposure (Markova et al., 2013). We have simulated this case using the human SimPops™ by decreasing the bosentan metabolism *V*_max_ values for both minor and major metabolites by 10-fold. This change led to eight simulated individuals out of 331 (2.42%) developing ALT elevations. DILIsym® correctly predicts the increased risk of toxicity due to this potential genetic polymorphism, though the predicted incidence rate is still less than the overall rate in the general population from the clinical study.

When bosentan was also simulated in the rat SimPops™, zero individual rats out of 191 developed ALT elevations. This is consistent with preclinical observations which have reported no toxicity in the rat (Leslie et al., 2007). It has been suggested that the difference in bile acid uptake inhibition by bosentan between rats and humans contributes to the species difference in hepatotoxicity (Ansede et al., 2010). In order to test this theory, we ran a simulation in the rat SimPops™ with uptake inhibition eliminated (*K*_*i*_ set to 1 × 10^10^); though bile acid accumulation in the liver was predicted (results not shown), no toxicity was observed. This suggests that the difference in the bile acid pool between humans and rats is likely a more significant contributor to the species difference in toxicity.

In order to ensure that the model can differentiate non-toxic BSEP inhibitors from toxic BSEP inhibitors, we modeled telmisartan in the human SimPops™. No toxicity was predicted in the human SimPops™, which is consistent with clinical observations. This was true whether telmisartan was modeled as a competitive or a noncompetitive inhibitor.

**Figure 7:**
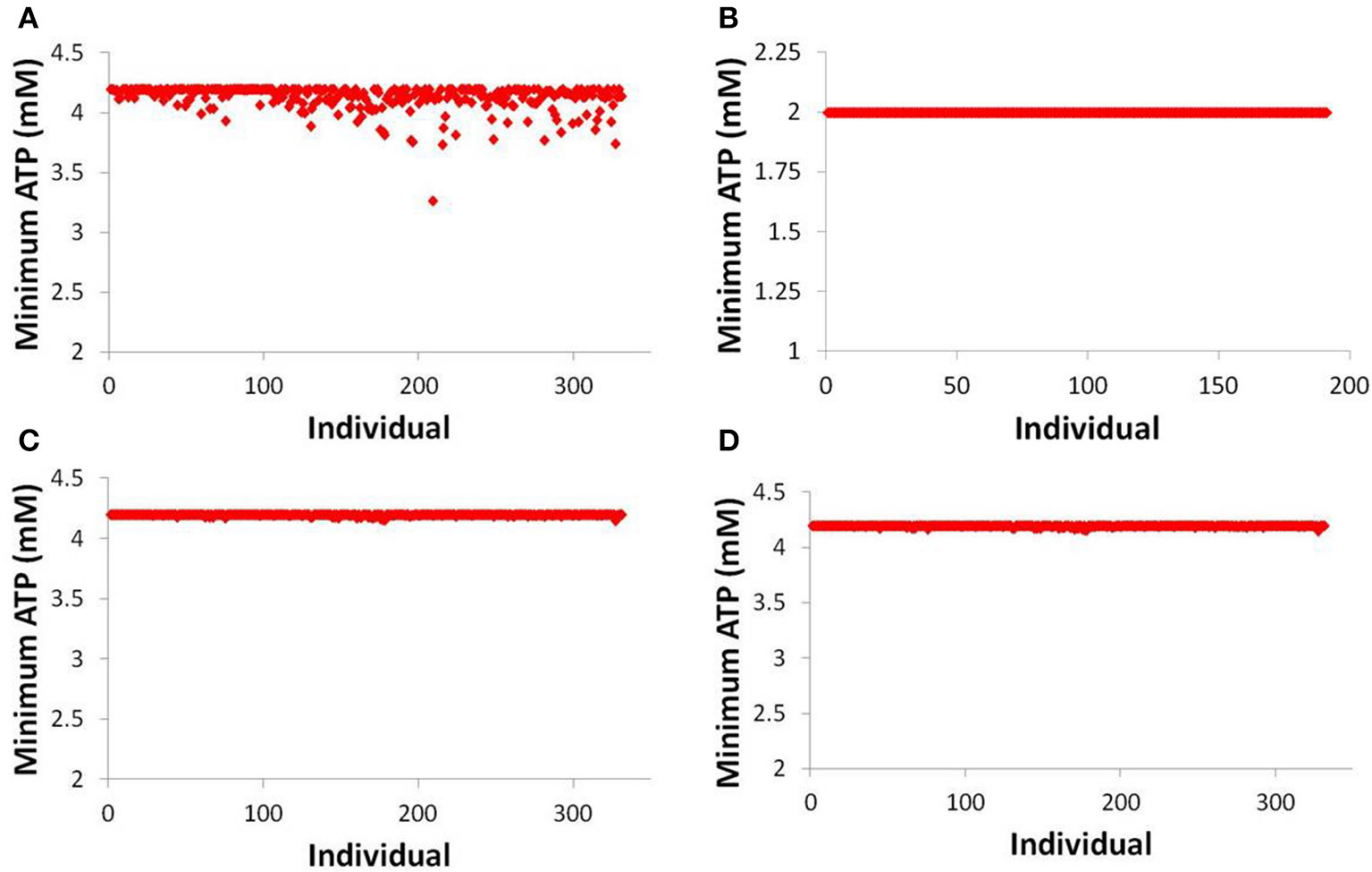
**ATP levels in individuals after simulated dosing of bosentan to the human SimPops™ (A) and the rat SimPops™ (B); and with simulated telmisartan dosing to the human SimPops™ when represented as a competitive (C) and noncompetitive (D) inhibitor**. Each point represents an individual within the human SimPops™; the baseline ATP concentration in humans is 4.2 mM, while in rats the baseline ATP concentration is 2.0 mM.

While there is scant difference between predicting one individual developing toxicity and predicting zero individuals developing toxicity (out of 331), a fuller understanding of the risk of a given compound can be gained by investigating more mechanistic data within DILIsym®. Figure 7 displays the minimum hepatic ATP for each individual in the human and rat SimPops™ for bosentan and the human SimPops™ for telmisartan (modeled as both a competitive and non-competitive inhibitor of BSEP). We can see that while only one simulated individual in the human SimPops™ had an ATP decline after bosentan dosing that ultimately led to toxicity, many more had ATP reductions visibly below the baseline value. This was not true of the rat, or of telmisartan in either case; the simulated individuals all have hepatic ATP values very close to the baseline value. This places the difference between bosentan and telmisartan, and the difference between rat and human, in sharper relief and demonstrates the potential of the model for predicting species differences in toxicity and differentiating between toxic and non-toxic BSEP inhibitors.

**Figure 8:**
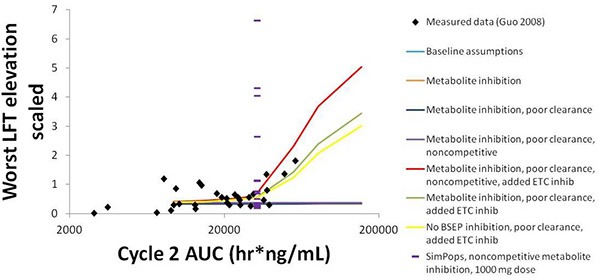
**Normalized LFT elevation at increasing exposure as measured by AUC_0-24_ on Day 1 of Cycle 2 (Day 22 overall) in the baseline human in DILIsym® and the clinical trial patients from Guo et al. (2008)**. The lines refer to the dose response simulated in DILIsym® under various assumed conditions listed in the table legend. “Baseline assumptions” refers to the case where only the parent CP-724,714 inhibits BSEP competitively and does not inhibit the electron transport chain (ETC). “Metabolite inhibition” refers to the case where the metabolite of CP-724,714 inhibits BSEP with the same *K*_*i*_ as the parent compound. “Poor clearance” refers to the case where the metabolite biliary clearance value is set to a value 10-fold lower than the parent biliary clearance value. “Noncompetitive” refers to the case where all BSEP inhibition is modeled as noncompetitive. “Added ETC inhibition” refers to the case where inhibition of the electron transport chain was simulated for both parent and metabolite. The points are individual patients from Guo et al. (2008). The purple dashes are the individuals in a human SimPops™.

The simulation results for CP-724,714 in the baseline human individual, and a comparison of these results to the clinical observations in Guo et al. (2008), are shown in Figure 8. The graph compares the normalized liver function test (LFT) elevation against the AUC of the drug in the patient's bloodstream on Day 22 (Day 1 of Cycle 2) of drug dosing, a measure of steady-state drug exposure. The normalized LFT elevation used by the Guo et al. (2008) paper to which our simulations are compared is given by the following expression (Guo et al., 2008):

$$\max\left(\frac{ALTfoldincrease}{5},\;\frac{bilirubinfoldincrease}{3}\right)$$

The simulation results are the maximum normalized LFT elevation at each dose simulated for each individual case. The graph shows that bile acid inhibition alone cannot explain the clinical toxicity, as liver function tests did not elevate in the simulations when bile acid inhibition alone was simulated. This was true for both competitive and noncompetitive inhibition. However, the inclusion of mitochondrial toxicity did, in fact, lead to a toxic response that was augmented when bile acid accumulation also occurred. The data are best described here by this combination of competitive inhibition and electron transport chain (ETC) inhibition.

The simulations also predict that both BSEP and mitochondrial ETC inhibition by CP-724,714's major metabolite are necessary to explain the toxicity, since CP-724,714 is rather rapidly metabolized by the liver and so parent residence time within the liver is somewhat limited. When the potential activity of the metabolite is not included, no toxicity is shown no matter the mechanism or combination of mechanisms selected. The accumulation of this metabolite is also necessary to explain the toxicity; a biliary clearance of 10% of the value for the parent compound was used in order to reproduce the clinical toxicity.

SimPops™ results for CP-724,714 in humans are also shown in Figure 8, with each individual in the SimPops™ represented by a purple dash. We found that while the simulated baseline individual did not display the toxicity that would have been expected from the clinical data, the population sample did contain several individuals who developed clinically-relevant ALT elevations, but only if the BSEP inhibition was noncompetitive. Furthermore, the range of injury in the SimPops™ is far wider than the range reported in the clinical study; the most severe normalized LFT elevation in our simulated population was 6.5, while the largest clinical normalized LFT in that exposure range was about 2. Twelve individuals in our 331-individual SimPops™, or 3.62%, developed toxicity; this is lower than the 36% of individuals in the exposure range near the simulated dose who developed LFT elevations. No individuals in the SimPops™ developed toxicity if the inhibition was competitive, demonstrating the importance of differentiating between modes of inhibition when determining BSEP inhibition constants.

**Figure 9:**
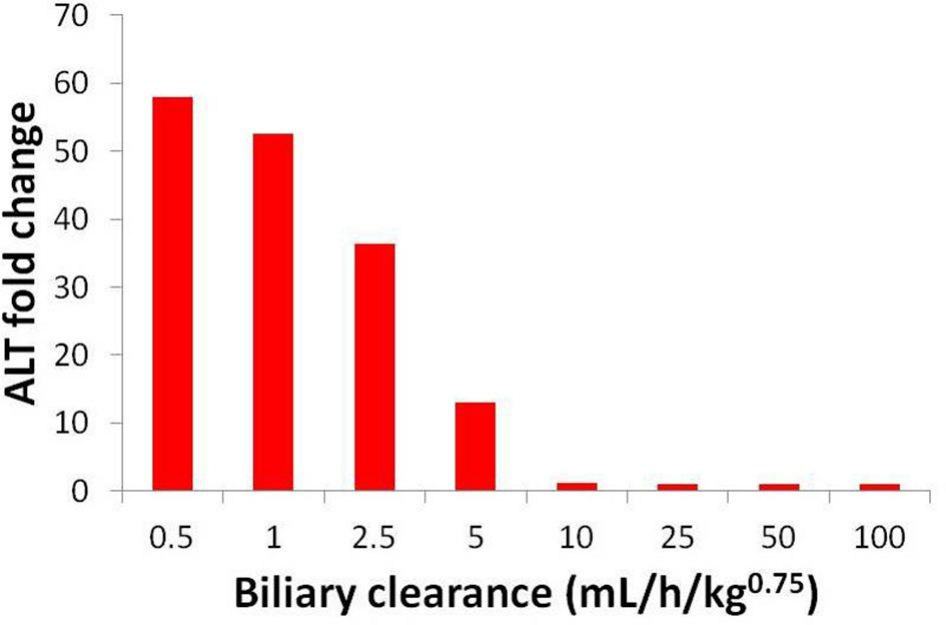
**ALT elevations at different values of metabolite biliary clearance given competitive BSEP inhibition and ETC inhibition**. The “poor clearance” cases explored in Figure 8 are represented here by the biliary clearance value of 5 mL/h/kg^0.75^.

Uncertainty about the biliary clearance of the CP-724,714 metabolite has an impact on simulated ALT elevations. Figure 9 shows simulated ALT elevations at a constant dose of CP-724,714 when the biliary clearance of the metabolite is modulated. This variable does not affect the plasma pharmacokinetics of parent CP-724,714; it is thus a degree of freedom in DILIsym®. However, as Figure 9 shows, it has a significant effect on the predicted toxicity of CP-724,714; DILIsym® suggests, therefore, that experiments clarifying the amount of hepatic accumulation and clearance of CP-724,714's major metabolite should be conducted in order to fully elucidate the molecule's toxicity.

## Discussion

We have used DILIsym® to model bosentan and found that, using the mechanistic simulation results calculating ATP decline, DILIsym® suggests that the potential for toxicity in a human population is greater than that in a rat population. Furthermore, the same ATP decline simulation results show a clear difference between the human population response to bosentan and to telmisartan. While the prediction of the toxic potential of bosentan was well below the actual clinical incidence rate, these results nevertheless show promise for the ability to use a mechanistic mathematical model of DILI, rather than small-animal models, to predict the human toxicity of a BSEP inhibitor. More revealingly, the modeling exercise suggested a potential reason for this discrepancy in toxicity between rats and humans. Previous work in this area demonstrated the difference in uptake transporter inhibition between rats and humans (Leslie et al., 2007; Ansede et al., 2010) and proposed that this was a contributing factor to the species difference. However, our simulations suggest that removing the uptake inhibition from the rat model altogether does not lead to liver toxicity in the rat. This suggests that the difference in bile acid pools and metabolic pathways are most likely the strongest contributor to the species difference in toxicity. The rat bile acid pool has more non-toxic bile acids, such as cholic acid and muricholic acid, and less of the toxic bile acids CDCA and LCA than the human (Hofmann, 2009; García-Cañaveras et al., 2012). Furthermore, the rat can hydroxylate LCA into the less-toxic hyodeoxycholic acid, which is a pathway that does not occur in the human (Hofmann, 2004). Because of these species differences in bile acid metabolism, our modeling questions the utility of current small-animal models in the prediction of BSEP inhibitor-mediated toxicity in humans.

Our results comparing the ATP decrement in the liver caused by bosentan and telmisartan demonstrate that DILIsym® can differentiate between a toxic and a non-toxic compound with a similar IC_50_. In the case of bosentan and telmisartan, the difference is mostly a pharmacokinetic one; telmisartan is dosed at a far lower dose than is bosentan. However, our results with increasing telmisartan dose suggest that a much higher dose of telmisartan, compared to bosentan, is necessary to cause toxicity in a human SimPops™. The suggestion is that dose is not necessarily predictive on its own; differences in metabolism and in liver uptake transporter affinity and capacity both are likely contributors to the differences in toxicity between bosentan and telmisartan.

While we qualitatively predict the species difference in bosentan toxicity and the toxicity of bosentan compared with the safety of telmisartan, we substantially underpredict the incidence rate of the toxicity of bosentan in the general population. Clinical trials predict 8–18% toxicity at the 1000 mg/day dosing level; DILIsym® predicted a much lower incidence rate even when individuals with metabolic polymorphisms were considered.

There are several possible reasons for this discrepancy, mostly related to the possibility of other mechanisms of toxicity not currently included in the DILIsym® model of bosentan. First, we do not represent the inhibition of basolateral bile acid transport by bosentan; this could prevent a potential clearance pathway for toxic bile acid species and lead to more toxicity. Recent research has shown that bosentan has the potential to inhibit MRP4, a basolateral bile acid transporter (Morgan et al., 2013). Exploratory simulations representing bosentan as a basolateral transporter inhibitor (reported in Table 2) suggest that this explanation is likely only part of the problem; if the bosentan basolateral transporter *K*_*i*_ is particularly low, however, this could account for some of the discrepancy in predicted incidence rates.

Second, DILIsym® does not represent bile acid toxicity in a mechanistic manner. Recent research suggests that the bile acids affect the mitochondria and potentially lead to the mitochondrial membrane permeability transition (Schulz et al., 2013); representing this effect in DILIsym® could introduce extra sources of variability and could lead to a higher predicted toxicity incidence rate. Indeed, work is underway in this area for DILIsym® and preliminary results suggest that the mechanistic model increases the predicted rate of bosentan toxicity without predicting toxicity in the rat; this work will be the focus of a subsequent paper.

Third, bosentan could cause toxicity through mechanisms not currently included in the model for bosentan or not currently represented in DILIsym®. Bosentan has been shown to inhibit phospholipid transport in rodents (Fouassier et al., 2002). Phospholipids protect the bile duct from the cytotoxic effect of bile acids; inhibition of phospholipid transport has been shown to lead to liver toxicity in the case of itraconazole (Yoshikado et al., 2011). While bosentan's mechanism of action is dissimilar to that of itraconazole, this shows that the ability of bosentan to interfere with phospholipid transport could be at least partially responsible for the observed toxicity of bosentan. Phospholipid transport and the toxic effect of its inhibition are not currently represented in DILIsym®; this is an area for potential future research and refinement.

Also, bosentan's effects on the mitochondria have not been elucidated. Previous research has shown that there is a correlation between the ability to inhibit BSEP and toxic effects on the mitochondria, and that this combined effect is itself correlated with DILI risk (Aleo et al., 2014). Furthermore, our simulations with CP-724,714 demonstrated that the toxicity seen in the clinic could not be explained without representing the drug's mitochondrial effects; while our research suggests that it is plausible that a compound could cause toxicity through bile acid transporter inhibition alone, it is also plausible that the toxicity of bosentan is due to a combined mitochondria/bile acid toxicity mechanism. Indeed, our modeling showed that this was most likely the case with CP-724,714. Research in this area is underway in our group and will focus on potential synergy between mitochondrial toxicity and bile acid buildup in the liver.

Fourth, DILIsym® does not represent the intracellular trafficking and potential localization of the drug within the hepatocyte. Further research in this area could help us improve DILIsym® and thus our prediction of toxicity incidence rates.

Our simulations with CP-724,714 demonstrate the ability of mechanistic modeling to consider and prioritize multiple hypotheses when modeling compounds where the cause of toxicity is not fully established. Potential variability in the degree of hepatic accumulation of a compound that inhibits BSEP is particularly important, as shown by Figure 9. This is an especially salient point to consider given that the toxicity of compounds such as troglitazone are suspected to be due to the hepatic accumulation of a BSEP-inhibiting metabolite (Masubuchi, 2006). Furthermore, the difference between competitive and non-competitive inhibition, the potential contribution of ETC inhibition to the observed toxicity, and the toxic potential of CP-724,714's metabolites are all sources of uncertainty that required a hypothesis-based approach to modeling. While this is of limited value for predicting toxicity on its own, the hypothesis-based modeling approach is potentially valuable in determining which experiments would be most impactful in using the model to properly predict toxicity. In the case of CP-724,714, these experiments include hepatic accumulation studies, BSEP inhibition studies, and ETC inhibition studies on CP-724,714's metabolites.

### Conflict of interest statement

The authors declare that the research was conducted in the absence of any commercial or financial relationships that could be construed as a potential conflict of interest.

## References

[B1] Aleo M. Luo Y. Swiss R. Bonin P. (2014). Human drug−induced liver injury severity is highly associated to dual inhibition of liver mitochondrial function and bile salt export pump. Hepatology 60, 1015–1022. 10.1002/hep.27206 24799086

[B2] Ansede J. H. Smith W. R. Perry C. H. St Claire R. L. 3rd. Brouwer K. R. (2010). An *in vitro* assay to assess transporter-based cholestatic hepatotoxicity using sandwich-cultured rat hepatocytes. Drug Metab. Dispos. Biol. Fate Chem. 38, 276–280. 10.1124/dmd.109.028407 19910518

[B3] Chatterjee S. Bijsmans I. T. G. W. van Mil S. W. C. Augustijns P. Annaert P. (2013). Toxicity and intracellular accumulation of bile acids in sandwich-cultured rat hepatocytes: role of glycine conjugates. Toxicol. Vitro Int. J. Publ. Assoc. 28, 218–230. 10.1016/j.tiv.2013.10.020 24211540

[B4] Dawson S. E. Stahl S. Paul N. Barber J. Kenna J. G. G. (2011). *In vitro* inhibition of the bile salt export pump correlates with risk of cholestatic drug induced liver injury in man. Drug Metab. Dispos. Biol. Fate Chem. 40, 130–138. 10.1124/dmd.111.040758 21965623

[B5] Dingemanse J. van Giersbergen P. L. M. (2004). Clinical pharmacology of bosentan, a dual endothelin receptor antagonist. Clin. Pharmacokinet. 43, 1089–1115. 10.2165/00003088-200443150-00003 15568889

[B6] Eriksson C. Gustavsson A. Kronvall T. Tysk C. (2011). Hepatotoxicity by bosentan in a patient with portopulmonary hypertension: a case-report and review of the literature. J. Gastrointest. Liver Dis. 20, 77–80. 21451802

[B7] Fattinger K. Funk C. Pantze M. Weber C. Reichen J. Stieger B.. (2001). The endothelin antagonist bosentan inhibits the canalicular bile salt export pump: a potential mechanism for hepatic adverse reactions. Clin. Pharmacol. Ther. 69, 223–231. 10.1067/mcp.2001.114667 11309550

[B8] Feng B. Xu J. J. Bi Y.-A. Mireles R. Davidson R. Duignan D. B.. (2009). Role of hepatic transporters in the disposition and hepatotoxicity of a HER2 tyrosine kinase inhibitor CP-724,714. Toxicol. Sci. 108, 492–500. 10.1093/toxsci/kfp033 19223659

[B9] Fouassier L. Kinnman N. Lefèvre G. Lasnier E. Rey C. Poupon R.. (2002). Contribution of mrp2 in alterations of canalicular bile formation by the endothelin antagonist bosentan. J. Hepatol. 37, 184–191. 10.1016/S0168-8278(02)00107-1 12127422

[B10] Funk C. Pantze M. Jehle L. Ponelle C. Scheuermann G. Lazendic M.. (2001). Troglitazone-induced intrahepatic cholestasis by an interference with the hepatobiliary export of bile acids in male and female rats. Correlation with the gender difference in troglitazone sulfate formation and the inhibition of the canalicular bile salt export pump (Bsep) by troglitazone and troglitazone sulfate. Toxicology 167, 83–98. 10.1016/S0300-483X(01)00460-7 11557132

[B11] García-Cañaveras J. C. Donato M. T. Castell J. V. Lahoz A. (2012). Targeted profiling of circulating and hepatic bile acids in human, mouse, and rat using a UPLC-MRM-MS-validated method. J. Lipid Res. 53, 2231–2241. 10.1194/jlr.D028803 22822028 PMC3435556

[B12] Guo F. Letrent S. P. Munster P. N. Britten C. D. Gelmon K. Tolcher A. W.. (2008). Pharmacokinetics of a HER2 tyrosine kinase inhibitor CP-724,714 in patients with advanced malignant HER2 positive solid tumors: correlations with clinical characteristics and safety. Cancer Chemother. Pharmacol. 62, 97–109. 10.1007/s00280-007-0579-4 17805538

[B13] Hofmann A. F. (2004). Detoxification of lithocholic acid, a toxic bile acid: relevance to drug hepatotoxicity. Drug Metab. Rev. 36, 703–722. 10.1081/DMR-200033475 15554243

[B14] Hofmann A. F. (2009). The enterohepatic circulation of bile acids in mammals: form and functions. Front. Biosci. 14, 2584–2598. 10.2741/3399 19273221

[B15] Howell B. A. Siler S. Q. Shoda L. K. M. Yang Y. Woodhead J. L. Watkins P. B. (2014). A mechanistic model of drug-induced liver injury AIDS the interpretation of elevated liver transaminase levels in a phase I clinical trial. CPT Pharmacomet. Syst. Pharmacol. 3, e98. 10.1038/psp.2013.74 24500662 PMC3944113

[B16] Howell B. A. Yang Y. Kumar R. Woodhead J. L. Harrill A. H. Clewell H. J. 3rd. (2012). *In vitro* to *in vivo* extrapolation and species response comparisons for drug-induced liver injury (DILI) using DILIsym™: a mechanistic, mathematical model of DILI. J. Pharmacokinet. Pharmacodyn. 39, 527–541. 10.1007/s10928-012-9266-0 22875368

[B17] Kis E. Ioja E. Nagy T. Szente L. Herédi-Szabó K. Krajcsi P. (2009). Effect of membrane cholesterol on BSEP/Bsep activity: species specificity studies for substrates and inhibitors. Drug Metab. Dispos. Biol. Fate Chem. 37, 1878–1886. 10.1124/dmd.108.024778 19520776

[B18] Kostrubsky S. E. Strom S. C. Kalgutkar A. S. Kulkarni S. Atherton J. Mireles R.. (2006). Inhibition of hepatobiliary transport as a predictive method for clinical hepatotoxicity of nefazodone. Toxicol. Sci. 90, 451–459. 10.1093/toxsci/kfj095 16410371

[B19] Lauer B. Tuschl G. Kling M. Mueller S. O. (2009). Species-specific toxicity of diclofenac and troglitazone in primary human and rat hepatocytes. Chem. Biol. Interact. 179, 17–24. 10.1016/j.cbi.2008.10.031 19022234

[B20] Leslie E. M. Watkins P. B. Kim R. B. Brouwer K. L. R. (2007). Differential inhibition of rat and human Na+-dependent taurocholate cotransporting polypeptide (NTCP/SLC10A1)by bosentan: a mechanism for species differences in hepatotoxicity. J. Pharmacol. Exp. Ther. 321, 1170–1178. 10.1124/jpet.106.119073 17374746

[B21] Mano Y. Usui T. Kamimura H. (2007). Effects of bosentan, an endothelin receptor antagonist, on bile salt export pump and multidrug resistance–associated protein 2. Biopharm. Drug Ldots 18, 13–18. 10.1002/bdd.527 17061295

[B22] Markova S. M. De Marco T. Bendjilali N. Kobashigawa E. A. Mefford J. Sodhi J.. (2013). Association of CYP2C9^*^2 with bosentan-induced liver injury. Clin. Pharmacol. Ther. 94, 678–686. 10.1038/clpt.2013.143 23863877 PMC3834031

[B23] Masubuchi Y. (2006). Metabolic and non-metabolic factors determining troglitazone hepatotoxicity: a review. Drug Metab. Pharmacokinet. 21, 347–356. 10.2133/dmpk.21.347 17072088

[B24] Meier Y. Pauli-Magnus C. Zanger U. M. Klein K. Schaeffeler E. Nussler A. K.. (2006). Interindividual variability of canalicular ATP-binding-cassette (ABC)-transporter expression in human liver. Hepatol. 44, 62–74. 10.1002/hep.21214 16799996

[B25] Ménochet K. Kenworthy K. E. Houston J. B. Galetin A. (2012). Simultaneous assessment of uptake and metabolism in rat hepatocytes: a comprehensive mechanistic model. J. Pharmacol. Exp. Ther. 341, 2–15. 10.1124/jpet.111.187112 22190645 PMC3310695

[B26] Meredith P. A. (1999). Optimal dosing characteristics of the angiotensin II receptor antagonist telmisartan. Am. J. Cardiol. 84, 7K–12K. 10.1016/S0002-9149(99)00400-2 10437738

[B27] Morgan R. E. van Staden C. J. Chen Y. Kalyanaraman N. Kalanzi J. Dunn R. T.. (2013). A multifactorial approach to hepatobiliary transporter assessment enables improved therapeutic compound development. Toxicol. Sci. 136, 216–241. 10.1093/toxsci/kft176 23956101

[B28] Munster P. N. Britten C. D. Mita M. Gelmon K. Minton S. E. Moulder S.. (2007). First study of the safety, tolerability, and pharmacokinetics of CP-724,714 in patients with advanced malignant solid HER2-expressing tumors. Clin. Cancer Res. 13, 1238–1245. 10.1158/1078-0432.CCR-06-1539 17317835

[B29] Schulz S. Schmitt S. Wimmer R. Aichler M. Eisenhofer S. Lichtmannegger J.. (2013). Progressive stages of mitochondrial destruction caused by cell toxic bile salts. Biochim. Biophys. Acta 1828, 2121–2133. 10.1016/j.bbamem.2013.05.007 23685124

[B30] Shoda L. K. M. Woodhead J. L. Siler S. Q. Watkins P. B. Howell B. A. (2014). Linking physiology to toxicity using DILIsym(®), a mechanistic mathematical model of drug-induced liver injury. Biopharm. Drug Dispos. 35, 33–49. 10.1002/bdd.1878 24214486

[B31] Smith M. T. (2003). Mechanisms of troglitazone hepatotoxicity. Chem. Res. Toxicol. 16, 679–687. 10.1021/tx034033e 12807350

[B32] Stangier J. Su C. A. Roth W. (2000). Pharmacokinetics of orally and intravenously administered telmisartan in healthy young and elderly volunteers and in hypertensive patients. J. Int. Med. Res. 28, 149–167. 10.1177/147323000002800401 11014323

[B33] Ubeaud G. Schmitt C. Jaeck D. (1995). Bosentan, a new endothelin receptor antagonist: prediction of the systemic plasma clearance in man from combined *in vivo* and *in vitro* data. Xenobiotica 25, 1381–1390. 10.3109/00498259509061925 8719912

[B34] Weber C. Schmitt R. Birnboeck H. Hopfgartner G. Marle S. P. V. Jones C. (1996). Pharmacokinetics and pharmacodynamics of the endothelin-receptor antagonist bosentan in healthy human subjects. Clin. Pharmacol. Ther. 60, 124–137. 10.1016/S0009-9236(96)90127-7 8823230

[B35] Woodhead J. L. Howell B. A. Yang Y. Harrill A. H. Clewell H. J. 3rd. Andersen M. E.. (2012). An analysis of N-acetylcysteine treatment for acetaminophen overdose using a systems model of drug-induced liver injury. J. Pharmacol. Exp. Ther. 342, 529–540. 10.1124/jpet.112.192930 22593093

[B36] Woodhead J. L. Yang K. Brouwer K. L. R. Siler S. Q. Stahl S. H. Ambroso J. L.. (2014). Mechanistic modeling reveals the critical knowledge gaps in bile acid-mediated DILI. CPT Pharmacomet. Syst. Pharmacol. 3, e123. 10.1038/psp.2014.21 25006780 PMC4120015

[B37] Yang K. Woodhead J. L. Watkins P. B. Howell B. A. Brouwer K. L. (2014). Systems pharmacology modeling predicts delayed presentation and species differences in bile acid-mediated troglitazone hepatotoxicity. Clin. Pharmacol. Ther. 96, 589–598. 10.1038/clpt.2014.158 25068506 PMC4480860

[B38] Yang K. Woodhead J. L. St. Claire R. Watkins P. B. Siler S. Q. Howell B. A. (2013). Quantitative relationship between intracellular lithocholic acid and toxicity in rat sandwich-cultured hepatocytes: incorporation into a mechanistic model of drug-induced liver injury. Toxicologist 132:226.

[B39] Yoshikado T. Takada T. Yamamoto T. Yamaji H. Ito K. Santa T.. (2011). Itraconazole-induced cholestasis: involvement of the inhibition of bile canalicular phospholipid translocator MDR3/ABCB4. Mol. Pharmacol. 79, 241–250. 10.1124/mol.110.067256 21056966

